# Water-Tree Resistant Characteristics of Crosslinker-Modified-SiO_2_/XLPE Nanocomposites

**DOI:** 10.3390/ma14061398

**Published:** 2021-03-13

**Authors:** Yong-Qi Zhang, Xuan Wang, Ping-Lan Yu, Wei-Feng Sun

**Affiliations:** 1 Key Laboratory of Engineering Dielectrics and Its Application, Ministry of Education, School of Electrical and Electronic Engineering, Harbin University of Science and Technology, Harbin 150080, China; jonegen@126.com; 2 Chaozhou Power Supply Bureau, Guangdong Power Grid Co. Ltd., Chaozhou 521000, China; kingstel@163.com

**Keywords:** crosslinked polyethylene, auxiliary crosslinker, nanodielectrics, water tree growth, dynamic thermo-mechanical analysis

## Abstract

Trimethylolpropane triacrylate (TMPTA) as a photoactive crosslinker is grafted onto hydrophobic nanosilica surface through click chemical reactions of mercapto double bonds to prepare the functionalized nanoparticles (TMPTA-s-SiO_2_), which are used to develop TMPTA-s-SiO_2_/XLPE nanocomposites with improvements in mechanical strength and electrical resistance. The expedited aging experiments of water-tree growth are performed with a water-knife electrode and analyzed in consistence with the mechanical performances evaluated by means of dynamic thermo-mechanical analysis (DMA) and tensile stress–strain characteristics. Due to the dense cross-linking network of polyethylene molecular chains formed on the TMPTA-modified surfaces of SiO_2_ nanofillers, TMPTA-s-SiO_2_ nanofillers are chemically introduced into XLPE matrix to acquire higher crosslinking degree and connection strength in the amorphous regions between polyethylene lamellae, accounting for the higher water-tree resistance and ameliorated mechanical performances, compared with pure XLPE and neat-SiO_2_/XLPE nanocomposite. Hydrophilic TMPTA molecules grafted on the nano-SiO_2_ surface can inhibit the condensation of water molecules into water micro-beads at insulation defects, thus attenuating the damage of water micro-beads to polyethylene configurations under alternating electric fields and thus restricting water-tree growth in amorphous regions. The intensified interfaces between TMPTA-s-SiO_2_ nanofillers and XLPE matrix limit the segment motions of polyethylene molecular chains and resist the diffusion of water molecules in XLPE amorphous regions, which further contributes to the excellent water-tree resistance of TMPTA-s-SiO_2_/XLPE nanocomposites.

## 1. Introduction

As the most important constituent of a power cable, the electrical insulation layer determines the transmission capacity and operating conditions of power system [1]. Crosslinked polyethylene (XLPE) with excellent heat and electrical resistances, as well as favorable mechanical performances, has been comprehensively applied to the insulation layer of power cables [2]. Ultraviolet (UV) initiation technique for producing XLPE suggests a strategy for the large-scale manufacturing of new type cables, which possesses the advantages of a high production rate, low cost in raw materials, minimal infrastructure investment, long continuous operation, and accurate controllability [3]. Photoactive auxiliary crosslinkers can improve the UV-initiated crosslinking reaction rate of polyethylene and ameliorate dielectric properties by introducing deep charge traps [4].

Water resistance is particularly required for special electrical power cables under a humid environment in submarine and tunnel engineering. Water molecules will gradually infiltrate XLPE insulation layer through structural defects to form water-trees under alternative current (AC) electric field, which will even transform into or trigger electrical-trees [5,6]. Although water-trees cannot immediately cause substantial damage to the insulation layer of power cables, it will continuously expand to increase dielectric loss and decrease electrical breakdown field until insulation failure, which degrades the cable life and the reliability of power supply [7,8]. Water-tree aging is one of the main factors leading to the deterioration of XLPE insulation. The formation and growth of water-trees rely not only on external factors, such as the strength and frequency of the applied electric field and operation time, but also on intrinsic features such as crystal morphology and additive content in insulating materials [9,10,11,12]. It is of great significance for the safe operation of power cable to improve the water-tree resistance of XLPE insulation materials.

According to the mechanism of water-tree growth in polymer materials, the mechanical stress produced by water molecules under AC electric field will impact polymer macro-molecular chains, and finally form tree-shaped micro-cracks filled with water molecules (as described by water-tree) in the amorphous region of polymer materials. XLPE and its composites are composed of crystal and amorphous regions in a semi-crystalline polymer structure. Water molecules will cause electrical stretch under an AC electric field and generate local pressures in XLPE-based materials, which gives rise to micro-cracking for water-tree growth [13]. Under the action of high-frequency electric field, the electrical and mechanical stresses from water micro-beads onto macro-molecular chains in amorphous regions lead to the fracture of XLPE molecules between polyethylene lamellae due to stress fatigue, by which water molecules will further infiltrate inter-lamellae micro-cracks to form the gradually expanding channels filled with condensed water molecules, as a macroscopic manifestation of the divergent water-tree development [14]. Water-knife electrode method could be used in accelerated water-tree aging experiments to successfully evaluate the water-tree resistance of XLPE insulating materials in cable insulation [15,16].

At present, effective schemes of improving the water-tree resistant performance of XLPE materials consist of polar-compound modification, filling dielectric nanoparticles (nanodielectrics), blending elastomer, and waterproof materials [17,18,19,20]. Although the modification with polar-compounds can reduce the development rate of water-trees, the considerable discrepancy in molecular polarity between them and XLPE matrix leads to the inevitable thermal migrations outward in the cable fabrication process and lacks of persistent high water resistance to withstand long time operation. The acceptable interfaces between silica (SiO_2_) nanoparticles and polymer matrix account for the improvements in mechanical and dielectric performances [21,22]. In the present study, we combine polar-compound modification with nanodielectric technology, not only to avoid thermal migrations of small crosslinker molecules out of polyethylene matrix, but also to chemically introduce SiO_2_ nanofillers into XLPE crosslinked network. This will effectively enhance molecular-chain interactions in the amorphous regions connecting crystal lamellae so as to achieve amelioration in mechanical properties and water-tree-resistant characteristics. Hydrophilic crosslinkers grafted onto the surfaces of SiO_2_ nanofillers are competent to restrict the formation of water micro-beads at insulation defects, contributing to the improvement in water-tree resistance.

## 2. Experimental Schemes

### 2.1. Materials Preparation

**Figure 1:**
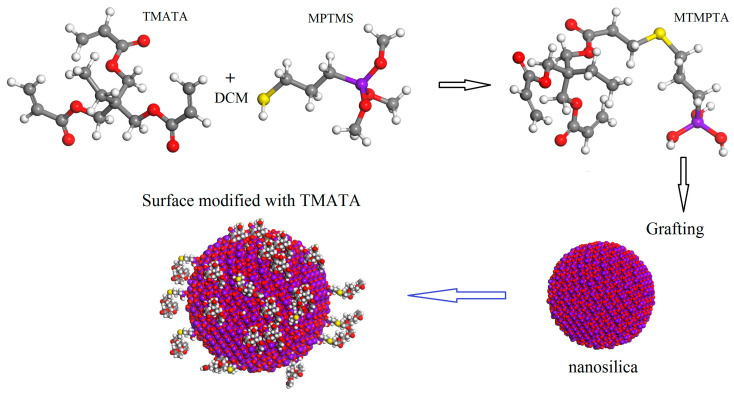
Schematic reactions of TMATA grafting onto nanosilica surface.

Raw materials and reaction mechanisms for synthesizing surface-modified nanosilica are listed in Table 1 and shown in Figure 1, respectively. Under magnetic stirring, 5.93 g TMPTA (0.02 mol) and 10 mL DCM are well-blended in a three-necked flask. The solution mixture of 3.92 g MPTMS (0.02 mol), 0.29 g TEA and 5 mL DCM, held in a constant-pressure drop funnel, is slowly dropped into the three-necked flask in ice-water bath under nitrogen protection. The obtained reaction solution is heated gradually to ambient temperature, held for 30 h, and then distilled in rotation under negative pressure to achieve the liquid product of MTMPTA in 82% yield. For grafting MTMPTA onto nanosilica surface (surface functionalization), 10 g nanosilica after drying treatment is suspended by ultrasonic dispersion method into a 100 mL aqueous/lanhydrous-ethanol (3:1) solution with a PH value of 4 adjusted by diluted hydrochloric acid (Jiangsu Heyuan Chemical Co. Ltd., Nanjing, China); the previously obtained liquid of MTMPTA is slowly dropped into a nanosilica liquid suspension and left for 8 h in nitrogen at 60 °C. The reaction product is washed with anhydrous ethanol, and then purified to extract out the prepared surface-modified nanoparticles from the mixed liquid by a centrifuge, which should be repeated three times. The obtained nanomaterial needs to be dried at 80 °C for 6 h in a vacuum oven to finally accomplish the preparation of the functionalized SiO_2_ nanoparticles with the surfaces grafted by TMPTA (TMPTA-s-SiO_2_).

Employing the UV-initiation crosslinking technique and melting blend method, XLPE and its composites filled with TMPTA-s-SiO_2_ (TMPTA-s-SiO_2_/XLPE nanocomposites) are prepared by blending the raw materials in mass percentages (wt%) as specified in Table 2, which are mixed in a Torque Rheometer (RM200C, Hapro Instruments Co. Ltd., Harbin, China) with a stirring speed of 60 rpm at 145 °C for 5 min. For UV-initiated crosslinking reactions, the prepared blend is pressed into a film specimen at 140 °C in a flat plate vulcanizer (XLB25-D, Hapro Instruments Co. Ltd., Harbin, China) with the pressure being increased by 5MPa per 5min from 0 to 15MPa, and then transferred into an UV-irradiation equipment (NVSU233A-U365, Riya Electronics Chemistry Co. Ltd., Shanghai, China) being irradiated for ~2 min by 365 nm UV light at normal pressure and room temperature in air atmosphere. After being short-circuit hot-degassed at 60 °C for 24 h in a vacuum oven to eliminate residual impurities and stresses, the pure XLPE and TMPTA-s-SiO_2_/XLPE nanocomposites are finally produced as individually named in Table 2. The reaction mechanism of UV-initiated polyethylene crosslinking can be found in Reference [23]. Through the UV-initiated crosslinking process, TMPTA-s-SiO_2_ nanofillers participate in crosslinking reactions and are introduced as the central node of the crosslinking network into the XLPE matrix.

### 2.2. Characterization and Testing Methodology

The type and components of hydrogen atoms (H) in MTMPTA samples are tested by nuclear magnetic resonance hydrogen spectrum (^1^H-NMR) to characterize the chemical structure of MTMPTA molecule. Molecular groups on the surfaces of TMPTA-s-SiO_2_ nanoparticles are analyzed with Fourier transform infrared spectrometer (FTIR-6100, Jiake Trading Co. Ltd., Shenyang, China). After being brittle-fractured in liquid nitrogen, the cross-sectional morphology of XLPE nanocomposites is observed by ultra-high-resolution scanning electron microscope (SU8020, Hitachi Co. Ltd., Tokyo, Japan) under cold field emissions to characterize the dispersion of nanofillers. Conforming to norm ASTM-D 2675-2011, solvent-extracted gel contents of XLPE and nanocomposite materials are tested to evaluate the crosslinking degree of XLPE matrix. According to the GB/T 1040.2-2006 norm, the stress–strain characteristics of “5A” dumbbell-shaped specimens of 1 mm thickness and 20 mm mark-distance are measured at a stretching speed of 5 mm/min. By means of dynamic thermo-mechanical analyzer (Q800DMA, TA apparatus Co. Ltd., Wilmington, DE, USA), viscoelastic characteristics of 40 × 10 × 1 mm^3^ cuboid specimens are analyzed at temperatures of −50~150 °C with a heating rate of 3 °C/min in nitrogen atmosphere, by specifying 0.375 N static and 0.3 N dynamic forces with the amplitude and frequency of 15 μm and 1 Hz, respectively.

**Figure 2:**
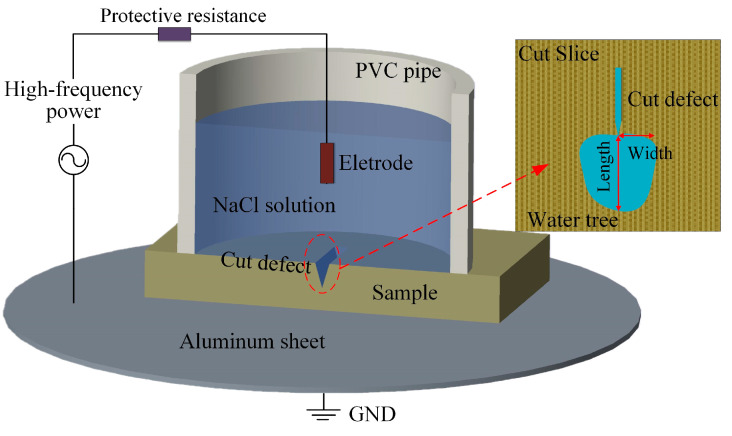
Schematic water-tree aging experiment with water-knife electrode method.

Water-tree growth experiments in an accelerated aging process are carried out with the water-knife electrode method, as shown in Figure 2. A conductive metal blade (knife electrode) of 0.01 mm curvature radius and 0.03 mm thickness is cut vertically by a depth of 1 mm into the cuboid samples of 5 mm in length and 2 mm in thickness to produce a blade-like concave (cut defect) at the edge of knife electrode. The 1.8 mol/L NaCl solution in PVC pipe is utilized as a water medium. To ensure sufficient probability of water-tree formation and represent observable tree-morphology, an AC high-voltage power supply with 4 kV effective voltage and 3.5 kHz frequency is adopted to carry out the aging test for 7 consecutive days. All specimens need to be pretreated in a vacuum environment for 40 min to eliminate residual air at blade edge. After water-tree growth has been accomplished, cuboid samples are cut longitudinally at cut defect points into slices of 120 μm thickness using manual rotary microtome (Leica RM2235, Chuangxun Medical Equipment Co. Ltd., Shanghai, China). Finally, slice samples are immersed into a methylene blue solution at 90 °C for 4 h, after which the legible water-tree morphology can be observed by optical microscope (SteREO Discovery.V20, Carl Zeiss AG, Germany).

## 3. Results and Discussion

### 3.1. Material Characterization

**Figure 3:**
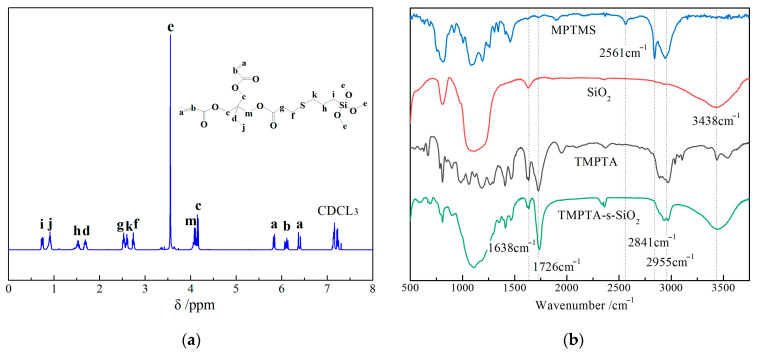
(**a**) Hydrogen nuclear magnetic spectrum of MTMPTA and (**b**) infrared transmission spectra of MPTMS, SiO_2_ nanoparticles, TMPTA, and TMPTA-s-SiO_2_ nanoparticles.

Characteristic peaks in the NMR-^1^H spectrum of MTMPTA arise at the chemical displacements (δ) in correct agreement with the theoretical values, as shown in Figure 3a. Molecular group of –CH=CH_2_ can be identified by H peaks at δ = 5.82, 6.38 and 6.09 ppm, while δ = 2.74 and 2.61 ppm peaks originate from the chemical displacement of H atoms on –CH_2_ group nearby S atoms, which proves the successful preparation of MTMPTA. Infrared transmission spectra of MPTMS, SiO_2_, TMPTA and TMPTA-s-SiO_2_ are shown in Figure 3b. Naked SiO_2_ nanoparticles present an infrared adsorption peak of Si-OH group at 3438 cm^−1^, while MPTMS (silane coupling agent) shows characteristic infrared peaks of –SH group at 2561 cm^−1^ and methyl/methylene group at 2955/2841 cm^−1^. TMPTA molecules exhibit double-bond vibration peaks of –CH_2_=CH_3_ and –C=O groups at 1638 cm^−1^ and 1726 cm^−1^, respectively. In comparison, the coincident multiple peaks of methyl, methylene, –CH_2_=CH_3_ and –C=O groups distinctly appear in the infrared spectrum of TMPTA-s-SiO_2_ nanoparticles, demonstrating that TMPTA (auxiliary crosslinker) has been successfully grafted onto nanosilica surface.

**Figure 4:**
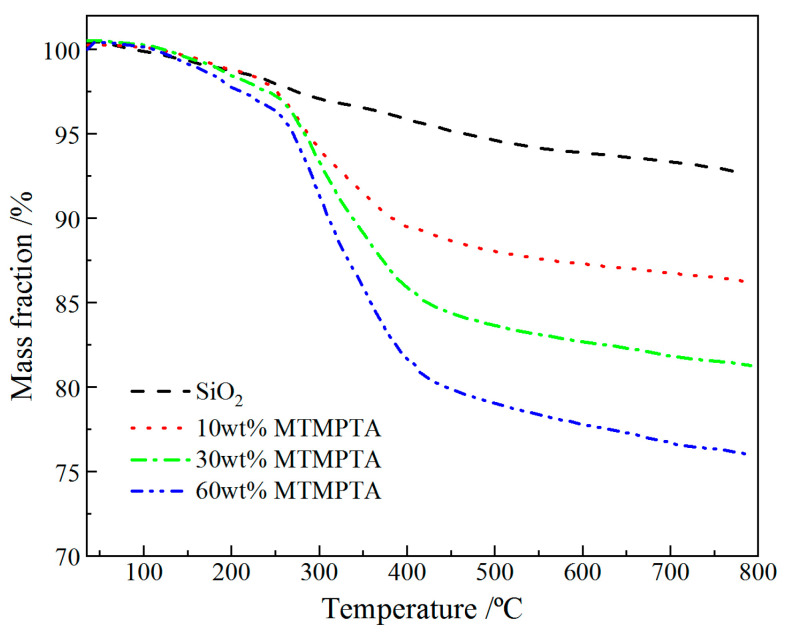
Thermogravimetric curves of TMPTA-s-SiO_2_ nanoparticles which have been prepared by adopting different contents of MTMPTA.

When the organically modified SiO_2_ nanoparticles are heated, the C–O bonds will break prior to Si–C bonds [24]. Accordingly, the weight losses of the modified nanoparticles at temperatures below 250 °C originate from thermal desorption of the physically adsorbed small molecules, in contrast to the weight losses at higher temperatures of 250~800 °C which are attributed to the decomposition of organic groups grafted on surfaces of SiO_2_ nanoparticles, as shown by thermogravimetric (TGM) curves of TMPTA-s-SiO_2_ nanoparticles in Figure 4. In consistence with ^1^H-NMR and infrared transmission spectra, TGM curves also provide a manifestation that TMPTA molecules have been chemically grafted onto nanosilica surfaces. In particular, it is found in surface modification processes that the grafting rate of TMPTA on surfaces of SiO_2_ nanoparticles can be fortified by increasing the dosage of MTMPTA. Nevertheless, considering the actual TMPTA concentration could be more easily controlled by altering the filling content of TMPTA-s-SiO_2_ nanoparticles, which is more feasible to ameliorate dielectric performances of XLPE materials, we chose the TMPTA-s-SiO_2_ nanoparticles modified with an intermediate dosage of 30 wt% MTMPTA to subsequently prepare TMPTA-s-SiO_2_/XLPE nanocomposites.

**Figure 5:**
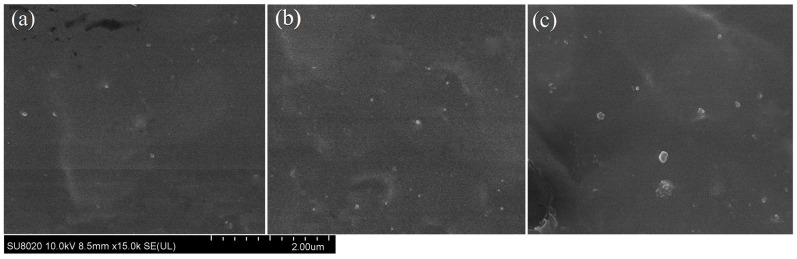
Cross-sectional SEM images: (**a**) 0.5wt%TMPTA-s-SiO_2_/XLPE, (**b**) 1.5wt%TMPTA-s-SiO_2_/XLPE and (**c**) 1.5wt%SiO_2_/XLPE nanocomposites.

Micro-morphology SEM images of TMPTA-s-SiO_2_/XLPE and SiO_2_/XLPE nanocomposites are shown in Figure 5. The 0.5 wt% and 1.5 wt% TMPTA-s-SiO_2_ nanofillers with sizes of 50~80 nm disperse well in XLPE matrix, persisting sufficient surface/volume ratios to effectively fulfill nanointerface functions in successful nanodielectrics, as shown in Figure 5a,b. In contrast, hydroxy groups on the naked surfaces of SiO_2_ nanoparticles have poor compatibility with XLPE matrix, leading to evident agglomerations of SiO_2_ nanofillers in 1.5wt%SiO_2_/XLPE nanocomposites, as manifested by larger nanofillers of >100 nm in Figure 5c. Through UV-initiation polyethylene crosslinking process, free radicals of TMPTA grafted onto nanosilica surfaces form chemical bonds with polyethylene molecular-chains, leading to the significant improvement of nanofiller dispersity in XLPE matrix.

### 3.2. Water-Tree Characteristics

**Figure 6:**
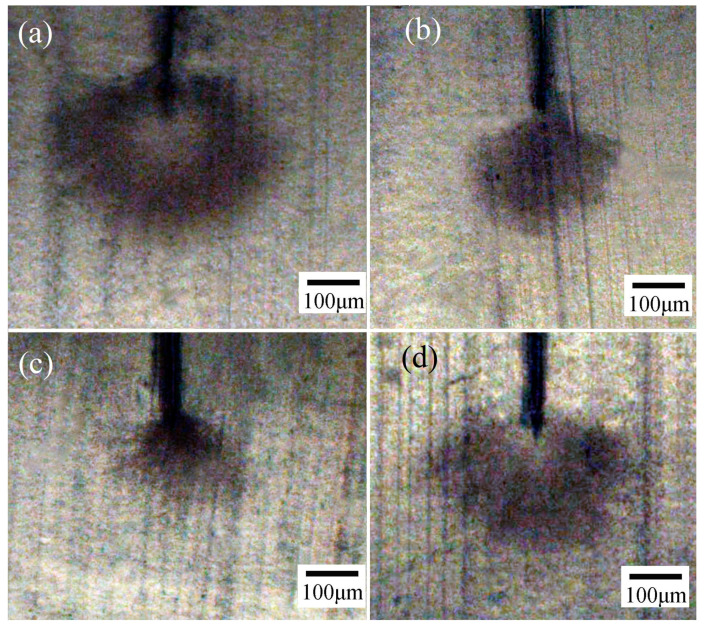
Water-tree morphology: (**a**) XLPE, (**b**) 0.5 wt%TMPTA-s-SiO_2_/XLPE, (**c**) 1.5 wt%TMPTA-s-SiO_2_/XLPE, (**d**) 1.5 wt% SiO_2_/XLPE.

Water-tree morphologies of XLPE and its nanocomposites are shown in Figure 6: the water-tree of XLPE grows emanatively around the cut defect tip, while TMPTA-s-SiO_2_/XLPE nanocomposites represent a uniform water-tree development with a significant lower growth speed (smaller size) tending toward ground electrode. By contrast, the restraint of water-tree growth in SiO_2_/XLPE nanocomposite is not such obvious as TMPTA-s-SiO_2_/XLPE nanocomposites. A higher degree of cross-linking will form a denser network of molecular-chains between polyethylene lamellae, which can impede the thermal diffusion of water molecules and withstand greater impact of water micro-beads under AC electric field, thus inhibiting water-tree growth. Hydrophobic surfaces of unmodified nanosilica also resist the thermal diffusion of water molecules and hereby reduce moisture absorption in XLPE matrix, resulting in a slight improvement in water resistance. The dense cross-linking network at the interface between TMPTA-s-SiO_2_ nanofillers and XLPE matrix limits the segment movement of polyethylene molecular-chains. Hydrophilic TMPTA groups distributing on surfaces of the well-dispersed TMPTA-s-SiO_2_ nanofillers in XLPE matrix can restrict water molecules from accumulating into water micro-beads, which evidently suppresses the formation and growth of water-trees. Therefore, 1.5wt%TMPTA-s-SiO_2_/XLPE nanocomposite acquires the most significant amelioration in water-tree resistance compared with XLPE and other nanocomposites.

According to the statistical dispersion of water-tree sizes shown in the morphology for identically repeated experiments, we use two-parameter Weibull statistics to evaluate water-tree structures by fitting experimental results as follows [22]:

(1) $$P(L)=1-\exp[-{\left(\frac{L}{L_{s}}\right)}^{\beta }]$$

in which *L* signifies the size of water-tree (length or width), *L_s_* denotes the characteristic water-tree size with a probability of 63.2%, shape parameter *β* characterizes the dispersion of experimental data, *P*(*L*) represents the failure probability of water-trees with a smaller size than *L*, which correlates with sampling capacity as expressed by

(2) $$P(L,n)\approx \frac{i-0.3}{n+0.4}\times 100\%$$

where *i* indicates the ascending order number of samples, and *n* denotes the total number of the tested samples, which is specialized as 10 for the present study.

**Figure 7:**
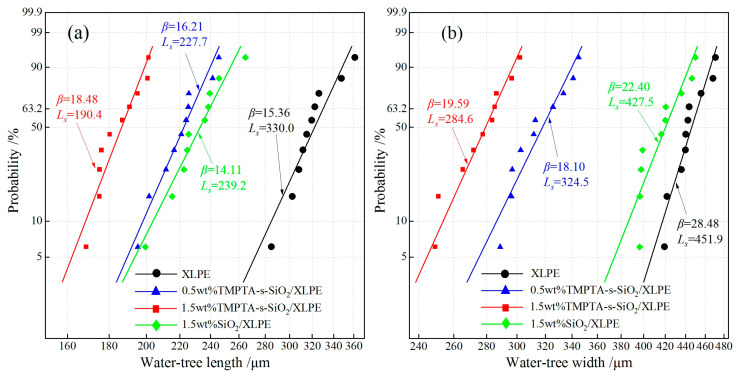
Water-tree characteristic (**a**) length and (**b**) width fitted in 2-parameter Weibull statistics.

Water-tree growth can be evaluated by two-parameter Weibull statistics of water-tree lengths and widths, as shown in Figure 7, indicating the characteristic value *L_s_* and shape parameter *β*. TMPTA-s-SiO_2_/XLPE nanocomposites show much smaller water-trees than XLPE. The characteristic dimensions of SiO_2_/XLPE and two TMPTA-s-SiO_2_/XLPE nanocomposites are reduced respectively by 19.6%, 25.7% and 73.4% for water-tree length, and decrease respectively by 14.0%, 50.2% and 58.8% for water-tree width, compared with that of XLPE. The appreciable improvement in the water-tree-resistant characteristics of TMPTA-s-SiO_2_/XLPE nanocomposites can be reinforced by increasing the filling content of TMPTA-s-SiO_2_ nanoparticles.

**Figure 8:**
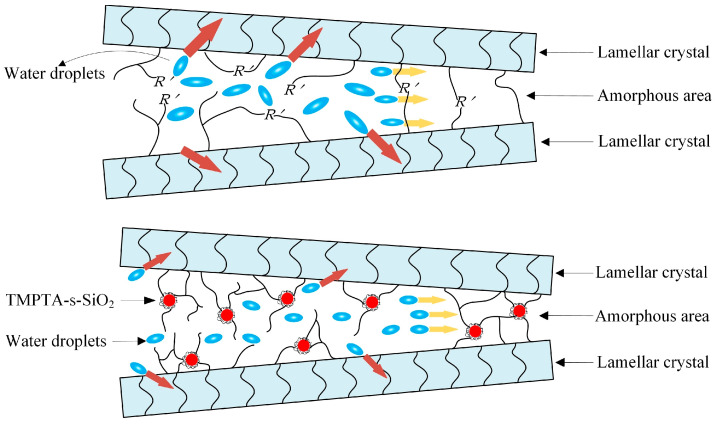
Schematic mechanism of water micro-beads impacting on amorphous regions under AC electric field in XLPE (**top panel**) and TMPTA-s-SiO_2_/XLPE nanocomposite (**bottom panel**).

Water micro-beads formed at polymer structure defects cause mechanical damage to the amorphous phase under AC electric field and destroy the molecular-chains connections between lamellae, which results in the extrusion and slip between adjacent lamellae and will form larger water-filled holes. As a result, the development of water-filled holes can be hindered by promoting the crosslinking density and connection strength of the molecular-chains between polyethylene lamellae. The molecular chains in amorphous phase of XLPE matrix bear mechanical stress from water micro-beads, as manifested by the macroscopic material tenacity, which should be enhanced to restrain water-tree development. The preferable compatibility of TMPTA with water can restrict water molecules from accumulating into micro-beads at polymer structure defects, thus reducing the damage from the impact of water micro-beads under AC electric field [25]. As schematically illustrated in Figure 8, TMPTA groups on surfaces of TMPTA-s-SiO_2_ nanofillers provide many chemical cross-linking points, which make TMPTA-s-SiO_2_ nanoparticles as central nodes being chemically introduced into XLPE crosslinking network. Therefore, a denser crosslinking structure with a great amount of the dispersive nanoscale pivots of network has been formed in XLPE amorphous phase, accounting for the crosslinking degree and mechanical strength of molecular-chains connecting adjacent polyethylene lamellae. Discriminated from the XLPE material prepared directly with TMPTA molecules that will be liable to evaporate out in crosslinking process, a much higher energy is required to break the composite structures between polyethylene lamellae in TMPTA-s-SiO_2_/XLPE nanocomposites [26]. Besides, the hydrophilic modified layer on TMPTA-s-SiO_2_ surface can disperse water micro-beads into smaller clusters of water molecules, thus reducing the mechanical stress of water micro-beads impacting on molecular-chains in amorphous regions under AC electric field.

### 3.3. Mechanical Performances

**Figure 9:**
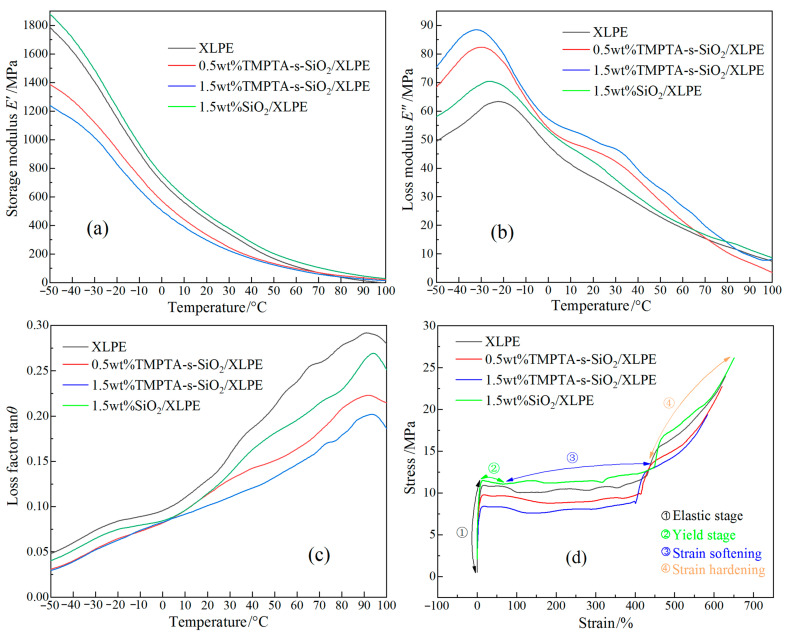
Dynamic thermo-mechanical spectra: (**a**) storage modulus, (**b**) loss modulus (**c**) loss factor, and (**d**) stress-strain characteristics of XLPE and its nanocomposites.

In dynamic relaxation temperature spectra, storage modulus *E*′ (elastic modulus) indicates the energy stored in elastic deformations and identifies the rigidity of polymer material, loss modulus *E*′′ indicates thermal energy loss caused by viscous deformations, and loss factor tan*θ* (*θ* denotes complex angle) indicates the ratio of loss to storage moduli which could be used to characterize material tenacity, as shown by the dynamic thermo-mechanical properties in Figure 9a–c. Table 3 lists the gel content (indicating crosslinking degree), the peak values of loss modulus *E*′ and loss factor tan*θ*, and the characteristic length and width of water-tree (*L*_s_ and *W*_s_). As the temperature rises, the kinetic energy of polyethylene molecular-chains increases, leading to the declining storage modulus (material rigidity). For all four materials, the storage modulus is increased for a higher XLPE crosslinking degree, as shown in Figure 9a, and the loss modulus increases at first, then decreases with increasing temperature, as shown in Figure 9b. The relaxation peak arising at −30 °C denotes the glass transition process, which is called β peak, deriving from the relaxation movements of XLPE molecular-chains in amorphous phase between polyethylene lamellae [27,28]. With the increase of crosslinking degree, β peak shifts towards a lower temperature and increases in peak value, implying the exacerbated relaxations of XLPE molecular-chains. The loss factor increases monotonously with increasing temperature until to 90 °C where a relaxation peak called α peak appears to characterize the mechanical relaxations caused by rotation and slip of the folded molecular-chains on lamella surface [29,30]. The crosslinking reaction can promote molecular-chain concentrations in amorphous regions between the polyethylene lamellae, and the formed crosslinking network will hinder the rotation and slip of the folded molecular-chains on lamella surface. TMPTA-s-SiO_2_ nanofillers act as connecting pivot nodes to essentially modify and enhance the crosslinking network of XLPE matrix. Hence, 1.5wt%TMPTA-s-SiO_2_/XLPE nanocomposite acquires the highest β relaxations and the lowest α relaxations.

As indicated in Table 3, crosslinking degree (gel content) is positive and negative in correlations with loss modulus and factor respectively. Accordingly, 1.5wt%TMPTA-s-SiO_2_/XLPE nanocomposite achieves the highest crosslinking degree and loss modulus, and the smallest loss factor, implying the most difficult molecular-chains relaxations between polyethylene lamellae. To this end, TMPTA-s-SiO_2_/XLPE nanocomposites can sustain more impact of water micro-beads under AC electric field, and hereby acquires a higher water-tree resistance compared with XLPE and SiO_2_/XLPE nanocomposite, which could be intensified by increasing TMPTA-s-SiO_2_ concentration.

Stress-strain characteristics manifesting in mechanical tensile process incorporate four stages: elastic, yield, strain softening and strain hardening, as shown in Figure 9d. Since crosslinking reactions cause the fracture of macro-molecular chains inside polyethylene materials [31,32], elastic yield strength decreases with the increase of crosslinking degree. Besides the increased density of molecular-chains connecting adjacent lamellae, TMPTA-s-SiO_2_ central nodes of divergent crosslinking network are formed in amorphous regions between polyethylene lamellae, which significantly improves the tensile strength of XLPE amorphous phase and limits the slip of polyethylene lamellae. Consequently, as manifested by the abated strain softening and elongated yield processes, TMPTA-s-SiO_2_/XLPE can resist higher impact of water micro-beads under AC electric field and inhibit the formation and growth of water-trees. When semi-crystalline polyethylene material is in strain hardening stage of tensile process, mechanical strain comes from the deformation of molecular-chains in amorphous regions. Therefore, 1.5wt%TMPTA-s-SiO_2_/XLPE nanocomposite represents the highest tensile gradient in strain hardening stage, confirming the significant improvements in molecular-chain density and tensile modulus of amorphous XLPE crosslinking network between lamellae.

## 4. Conclusions

Auxiliary crosslinker TMPTA has successfully been grafted onto nano-SiO_2_ surfaces by click chemical reactions of mercapto double-bonds, based on which UV-initiated XLPE nanocomposites are developed to ameliorate water-tree resistant characteristics for submarine cable fabrications. By means of water-knife electrode method, the accelerated water-tree aging experiments are carried out to investigate the modification mechanism of nano-SiO_2_ functionalized with auxiliary crosslinker to inhibit water-tree growth in XLPE matrix. The formation and development of water-trees are analyzed in coordination of XLPE crosslinking degree, stress-strain characteristics and dynamic thermo-mechanical performance. XLPE crosslinking degree is positive and negative in correlations with loss modulus and loss factor/tensile strength, respectively. Water-tree resistance of crosslinker-modified-SiO_2_/XLPE nanocomposites could be further promoted by increasing nanofiller concentration. Nano-SiO_2_ with the surface being functionalized by TMPTA can be introduced as central nodes into XLPE crosslinking network to increase XLPE crosslinking degree, molecular-chain density and tensile modulus in amorphous phase between polyethylene lamellae, which means an improved capability of resisting mechanical impacts of water micro-beads under AC electric field. Hydrophilic TMPTA grafted on nanosilica surfaces will restrain water molecules from condensing into water micro-beads, which reduces the mechanical stress caused by micro-water beads under AC electric field onto the amorphous regions between polyethylene lamellae, contributing to the improvement of water-tree characteristics. The present study suggests a strategy of combing chemical grafting and nanodielectric technologies to ameliorate water resistance of UV-initiated XLPE.

## Figures and Tables

**Table 1:** Raw materials for preparing TMPTA surface-modified SiO_2_ nanoparticles.

Materials	Source	Model
Linear low density polyethylene (LLDPE)	Jilin Petrochemical China Petro Co., Ltd., Changchun, China	DFDA 7042
4-hydroxy benzophenone laurate	Harbin University of Science and Technology, Harbin, China	——
Trimethylolpropane triacrylate (TMPTA)	Maklin Biochemical Technology Co. Ltd., Shanghai, China	——
SiO_2_ nanoparticles (~40 nm in diameter)		
3-mercaptopropyl trimethoxysilane (MPTMS)	Jiangsu Heyuan Chemical Co. Ltd., Nanjing, China	——
Dichloromethane (DCM)	Fuyu Fine Chemical Co. Ltd., Tianjin, China	Analytical purity
Triethylamine (TEA)		
Anhydrous ethanol (EtOH)		

**Table 2:** Blending components (wt%) of raw materials for preparing XLPE and its nanocomposites.

Materials	LLDPE	BPL	TMPTA	TMPTA-s-SiO_2_	SiO_2_
XLPE	96.7	2	1	0	0
0.5wt%TMPTA-s-SiO_2_/XLPE	97.2	2	0	0.5	0
1.5wt%TMPTA-s-SiO_2_/XLPE	96.2	2	0	1.5	0
1.5wt%SiO_2_/XLPE	95.2	2	1	0	1.5

**Table 3:** Crosslinking degree (gel content), ambient viscoelasticity (loss modulus *E*” and factor tan*θ*), and water-tree dimension (characteristic length *L*_s_ and width *W*_s_).

Samples	Gel Content/%	*E*”/MPa	tan*θ*	*L*_s_/μm	*W*_s_/μm
Pure XLPE	70.6	68	0.072	330.02	451.9
0.5wt%TMPTA-s-SiO_2_/XLPE	73.2	72	0.055	227.7	324.5
1.5wt%TMPTA-s-SiO_2_/XLPE	88.1	79	0.050	190.35	284.6
1.5wt% SiO_2_/XLPE	66.4	65	0.082	239.2	427.5

## Data Availability

Experimental methods and results are available from the authors.
